# High MIG-6 expression promotes tumor proliferation and metastasis of gastric cancer

**DOI:** 10.7150/jca.98431

**Published:** 2025-04-21

**Authors:** Wenqiu Zhao, Tao Jin, Yun Liu, Shihe Shao, Feilun Cui

**Affiliations:** 1 Department of Clinical Laboratory, Xuzhou Traditional Chinese Medicine Hospital Affiliated to Nanjing University of Chinese Medicine, China.; 2 The Affiliated Taizhou Second People's Hospital of Yangzhou University, China.; 3 Yixing People's Hospital, China.; 4 School of Medicine, Jiangsu University, China.

**Keywords:** Gastric cancer, EGFR, MIG-6, metastasis, proliferation, survival.

## Abstract

**Background:** Mitogen-inducible gene-6 (*MIG-6*) is a feedback inhibitor that targets activated epidermal growth factor receptor (EGFR) and suppresses tumor growth fueled by constitutively activated EGFR. Nevertheless, the action mechanism of *MIG-6* in gastric cancer (GC) remains to be elucidated.

**Methods:** Western blotting, fluorescence quantitative PCR, and immunohistochemistry were performed to detect the expression of *MIG-6* in GC cell lines and tissues. Public databases were used to analyze *MIG-6* in patients with GC. Furthermore, the GC cell lines were selected for the knockdown and overexpression of *MIG-6*.

**Results:** Bioinformatics and histological analyses showed that *MIG-6* was elevated in human GC tissues and cells. The Kaplan-Meier plotter showed that patients with elevated MIG-6 expression had significantly shorter survival. Furthermore, small interference RNA-mediated reduction of *MIG-6* expression decreased EGFR/AKT signaling, as well as the proliferation and metastasis of human GC cells *in vitro*, whereas its overexpression increased these actions. Also, *MIG-6* overexpression promoted the epithelial-mesenchymal transition of GC cells as well as the expression of the migration-associated protein matrix metalloproteinase-9 *in vitro*.

**Conclusion:** These results suggest that *MIG-6* can serve as a new prognostic biomarker or potential therapeutic target for the identification of patients with poor survival.

## 1. Introduction

The global incidence of gastric cancer (GC), a malignant tumor of the gastric mucosa, and the mortality associated with the disease rank fifth and fourth in human cancers, respectively 1. High salt intake, a diet poor in fruits and vegetables, ethnicity, heredity, and *Helicobacter pylori* infection are the risk factors for the illness 2. Unfortunately, the early stages of the disease are often asymptomatic, and at the time of surgery, approximately 60% of the patients with GC have locally progressed and metastatic illness. Surgical resection has a very low therapeutic efficacy 3, 4. Therefore, identifying new biomarkers and investigating the molecular mechanisms of metastasis and proliferation for the diagnosis and treatment of GC require urgent attention.

Mitogen-inducible gene-6 (*MIG-6*), also referred to as ERBB receptor feedback inhibitor 1 (*ERRFI1*), receptor-associated late transducer (*RALT*), or gene 33, is an immediate early response gene that can be directly activated by several mitogens and common chronic stress stimuli, such as hypoxia, hormones, and growth factors (such as epidermal growth factor) 5. *MIG-6* encodes a scaffolding adaptor protein that interacts with other signal molecules to influence signal transduction. *MIG-6* can selectively target activated epidermal growth factor receptor (EGFR) and act as an EGFR feedback inhibitor, which indicates its critical role in human cancers. Additionally, studies have revealed that *MIG-6* inhibits EGFR signaling on two different levels: by binding to the kinase domain and decreasing EGFR catalytic activity and by causing EGFR molecules to be internalized and eventually degraded 6, 7. For instance, Maity and Reschke 8, 9 observed that suppressing the expression of *MIG-6* prolongs the activation of the EGFR signaling pathway and promotes the genesis and development of malignancies, including lung and liver tumors. According to research by Ying et al. 10, *MIG-6* alleviates the malignant potential of glioblastoma multiforme cells by promoting EGFR trafficking into late endosomes/lysosomes and encouraging its destruction. Furthermore, in lung cancer and glioblastoma in humans, *MIG-6* is genetically altered and transcriptionally repressed but not in other malignancies 9, 10. Although several studies have shown that *MIG-6* is a tumor suppressor gene in human malignancies, mounting evidence suggests that *MIG-6* plays contradictory roles in growth regulation and tumor progression. He et al. 11, for instance, noted that *MIG-6* controls an EGFR-independent pathway to stimulate the proliferation of triple-negative breast cancer cells. Additionally, Kang et al. 12 claimed that *MIG-6* overexpression accelerates cell proliferation, invasion, and epithelial-mesenchymal transition (EMT) in EGFR-directed tyrosine kinase inhibitor (TKI)-resistant lung cancer cells compared with EGFR-TKI sensitive cells. These findings allude that *MIG-6* is a context-dependent regulator in malignancies and that the connection between *MIG-6* and EGFR is extremely complex. Thus, the role of *MIG-6* in GC remains unclear.

Here, our bioinformatics and histological analyses uncover that MIG-6 is upregulated in GC and that MIG-6 upregulation is positively correlated with poorer clinical outcomes in GC. Moreover, *MIG-6* overexpression increased EGFR/AKT signaling and promoted tumor proliferation and metastasis of GC cells *in vitro*. Also, *MIG-6* overexpression induces EMT in GC cells. These results imply a hitherto unreported tumor-promoting role of *MIG-6* in GC. Overall, our results showed that *MIG-6* could regulate GC and may serve as a potential target for antineoplastic therapies.

## 2. Materials and Methods

### 2.1 Patients and samples

This study was approved by the Ethics Committee of the School of Medicine, Jiangsu University, and informed consent was obtained from all patients. A total of 53 surgical specimens of GC were randomly selected between 2015 and 2016 from the General Surgery Department of the First People's Hospital of Zhenjiang, Jiangsu, China. Inclusion criteria: patients who had not received any form of anti-tumor therapy, such as chemoradiotherapy before surgery, and were diagnosed with GC after a pathological diagnosis. Exclusion criteria: patients with a family history of GC and those who had received radiotherapy or chemotherapy. The samples were acquired from the tumor tissue (T, avoiding tumor necrotic region) and the matching paracancerous tissue (P, distance from tumor focus >5 cm). The Cancer Staging Manual, seventh edition, of the American Joint Committee on Cancer was used to determine the participants' tumor stages.

### 2.2 Immunohistochemistry

The gastric tumor tissues were serially sectioned at a thickness of 4 μm and embedded in paraffin. Hydrogen peroxide (3%) was used to block endogenous peroxidase activity for 10 min, and bovine serum albumin (5%, Boster Bioengineering, Wuhan, China) was used to block the slides. The tissue sections were incubated with primary antibodies MIG-6 (Cell Signaling Technology, 1:300) overnight at 4°C. The sections were subsequently incubated overnight in a humidified chamber at 4°C, visualized with 3,3 diaminobenzidine, and counterstained with hematoxylin and eosin for microscopic examination.

MIG-6 protein expression in the cytoplasm of tumor cells was evaluated by measuring the intensity of staining and the proportion of positively stained cells. The scoring system was as follows: For positive cells that stained yellowish, light-brown, or dark-brown, a score of 1-3 was given; otherwise, a score of 0 was awarded.

### 2.3 Cell culture

Human gastric epithelial cells GES-1 and the four human GC cell lines (BGC-823, SGC-7901, MGC-803, and AGS) were sourced from our laboratory. Fetal bovine serum (FBS, Gibco, Grand Island, NY, USA)-supplemented F12 medium (Gibco) was used to culture the AGS cells, and RPMI-1640 medium (Gibco) supplemented with 10% FBS was used to culture the other cancer cell lines. All cells were cultured in a humid incubator at 37°C with 5% CO_2_.

### 2.4 Plasmid construction and small interference RNA (siRNA) and cell transfection

The pcDNA-*MIG-6* plasmids and vector plasmids were purchased from Bioworld Company (Nanjing, China). Lipofectamine 2000 (Invitrogen, Shanghai, China) was used to transfect the BGC-823 cells with the plasmids. Western blotting was performed to evaluate the efficiency of the overexpression. The siRNA-targeted *MIG-6* procured from GenePharma Company (Shanghai, China) was transfected into SGC-7901 cells for downregulating the expression of *MIG-6* in GC cells.

### 2.5 RNA extraction and real-time PCR analysis

Total RNA was extracted from paraffin sections of GC tissues using TRIzol reagent according to the manufacturer's instructions (Invitrogen, Life Technologies Corporation, CA, USA), and cDNA was synthesized using the HiScript® QRT SuperMix in the qPCR Kit (Vazyme, Nanjing, China). The mRNA levels of *MIG-6* were evaluated using the Applied Biosystems Real-Time PCR System. The sequences of the qRT-PCR primers were as follows: *MIG-6*: 5′-ATAGAAGATGGTCAGCAGAAG-3′, *MIG-6*-reverse: 5′-CATTGAGGTAAGACGGAAGG-3′. The 2^-∆∆CT^ relative quantitative approach was used to quantify the data, and glyceraldehyde 3-phosphate dehydrogenase (GAPDH) was employed as the internal control.

### 2.6 Western blotting

Phenylmethanesulfonyl fluoride and phosphatase inhibitors were added to radioimmunoprecipitation assay lysis buffer (Beyotime Biotechnology, Shanghai, China), and the mixture was used to lyse cell proteins. An equal amount of proteins (100 μg) was then separated using SDS-PAGE (12%) and transferred to polyvinylidene fluoride membranes. Before being incubated with primary antibodies overnight at 4°C, the membranes were blocked with 5% nonfat dry milk powder for 2 h at room temperature. The enhanced chemiluminescence system (Image Quant LAS 4000 mini, USA) was used to view the membranes after applying the appropriate secondary antibody at room temperature for 2 h. The relative densities of the bands were measured using ImageJ (http://rsb.info.nih.gov/ij/). The relative protein levels were calculated using the loading control GAPDH. Each test was repeated at least three times.

### 2.7 Transwell migration assay

CoStar transwell chambers (8 m pore size, Corning, NY, USA) were used for the transwell migration tests, which were performed with 1 × 10^5^ cells per well. Cells were seeded in the top chambers of the wells using 300 μL of serum-free medium to promote cell migration, whereas the lower chambers were filled with 600 μL of medium containing 10% FBS. A cotton swab was used to scrape out the cells on the upper side of the membrane during 24 h of incubation at 37°C and 5% CO_2_. The cells that had moved to the bottom surface of the membrane were stained with crystal violet after being fixed with 4% paraformaldehyde. The cells were photographed and counted in five arbitrary fields.

### 2.8 Cell proliferation assay

Following the manufacturer's instructions, the cell counting kit-8 (CCK-8 kit, Tongren, Shanghai, China) was used to assess the proliferative ability of BGC-823 and SGC-7901 cells. In 96-well plates, 1 × 10^3^ transfected cells were planted and cultured. CCK-8 solution (10 μL) was then added to each well before being incubated for 1 h. At different time intervals (24 h, 48 h, and 72 h), the absorbance at 450 nm was measured using a microplate reader.

### 2.9 Colony-formation assay

Colony-formation experiments were performed in triplicate on six-well plates with 1,000 cells per well. The cells were cultivated in a humidified incubator with 5% CO_2_ at 37°C. When discernible clones developed in the wells after 14 d, culturing was stopped, and the cells were fixed with 4% paraformaldehyde, and stained with crystal violet.

### 2.10 Statistical analyses

GraphPad 8.0 was used for all statistical analyses. The student's t-test was used to identify whether significant differences were present between groups. Fisher's exact test was used to determine the relationship between *MIG-6* expression and clinicopathological variables. Overall survival curves were produced using the Kaplan-Meier technique, and the log-rank test was used for comparison and Fisher's exact test for estimation. Statistical significance was considered as a P value of <0.05. All tests were conducted at least three times.

## 3. Results

### 3.1 MIG-6 expression in GC tissues is positively correlated with disease progression and poor prognosis

**Figure 1:**
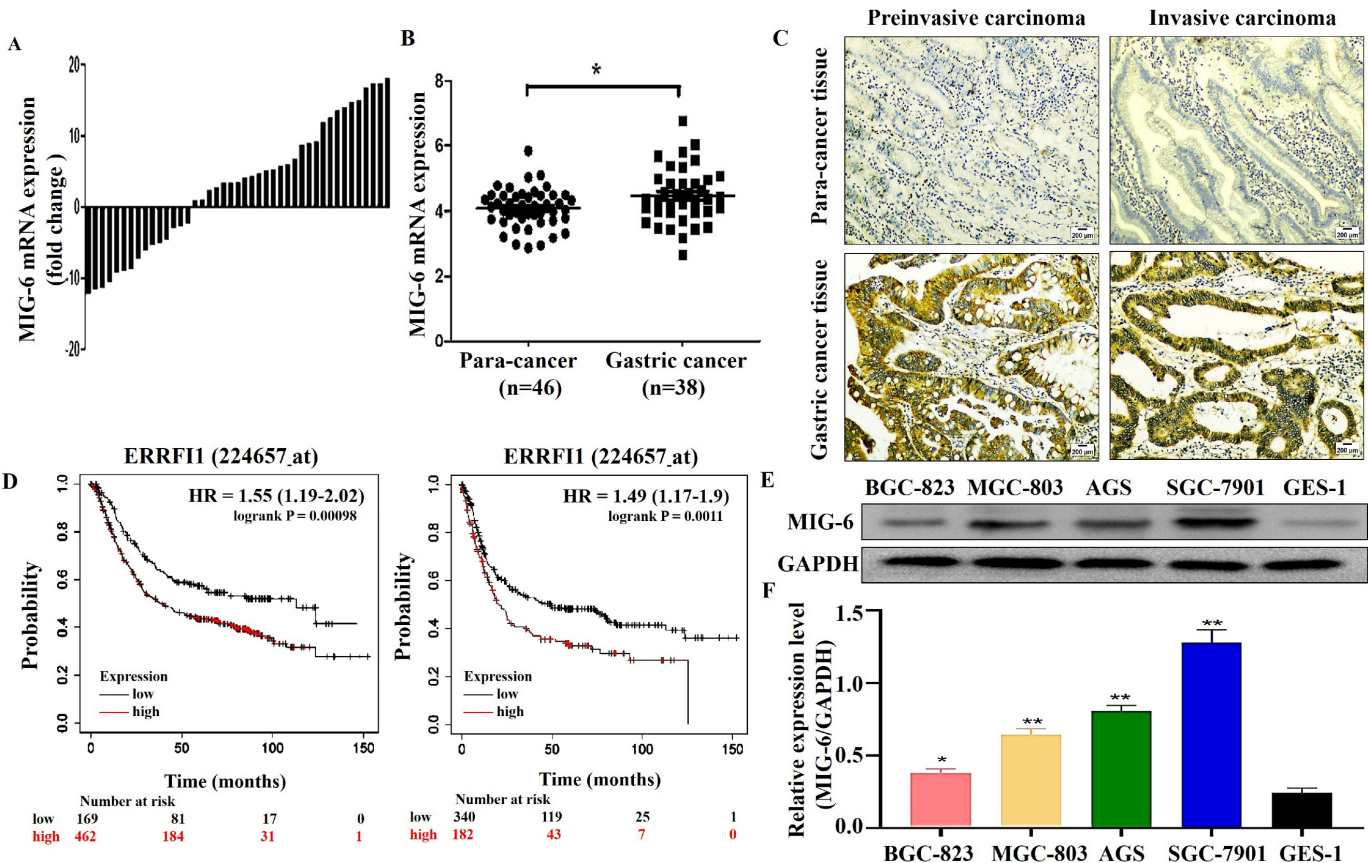
The expression of MIG-6 in the GC tissues and cell lines. **(A)** The mRNA level of MIG-6 in 43 pairs of GC tissues and paracancerous tissues was detected by qRT-PCR. **(B)** The mRNA level of MIG-6 in the paracancerous tissues (n = 38) and GC tissues (n = 46) from the ONCOMINE database. **(C)** Immunohistochemistry detected the protein level of MIG-6 in the paracancerous tissues (left panel) and GC tissues (right panel) (Original magnification: ×100). **(D)** The effect of MIG-6 mRNA expression on the overall survival and progression-free survival in GC patients was analyzed by Kaplan-Meier Plotter (http://www.kmplot.com). **(E)** and **(F)** The expression of MIG-6 in human gastric epithelial cells GES-1 and GC cell lines were detected by western blotting. *P < 0.05, **P < 0.01

To investigate *MIG-6* expression in GC, *MIG-6* mRNA levels in GC tissues and paracancerous tissues were determined using qRT-PCR. The findings indicated that among the 43 paraffin sections of the GC tissues, the *MIG-6* mRNA level was elevated in 65.1% (28/43) of the tissues (Figure 1A), which agrees with the analysis of the ONCOMINE dataset (Figure 1B) (https://www.oncomine.org). Immuno-histochemical examination of *MIG-6* protein expression in 53 pairs of tumor tissues and paracancerous tissues revealed that the expression was positive in GC tissues (Figure 1C). In addition, *MIG-6* expression was considerably higher in the cancer tissues of 64.2% (34/53) patients than in matching paracancerous tissues. Moreover, high *MIG-6* expression was positively correlated with higher differentiation (P = 0.02) and advanced clinical stage (P = 0.02) in patients with GC (Table 1). Furthermore, the Kaplan-Meier plotter signified that *MIG-6* protein expression was inversely correlated with overall survival and progression-free survival in patients with GC (Figure 1D), indicating that *MIG-6* may be a predictive biomarker for poor prognosis. As this result differs from those from previous publications on *MIG-6*, the following functional and molecular testing studies were performed.

### 3.2 The expression of MIG-6 differs in normal gastric mucosal epithelial cells and human GC cell lines

*MIG-6* was expressed in both GES-1 and GC cells. However, compared with GES-1 cells, the expression was higher in GC cells. The expression was the lowest in BGC-823 cells and the highest in SGC-7901 cells (Figure 1E).

### 3.3 MIG-6 regulates EGFR activation and signaling in GC cells

**Figure 2:**
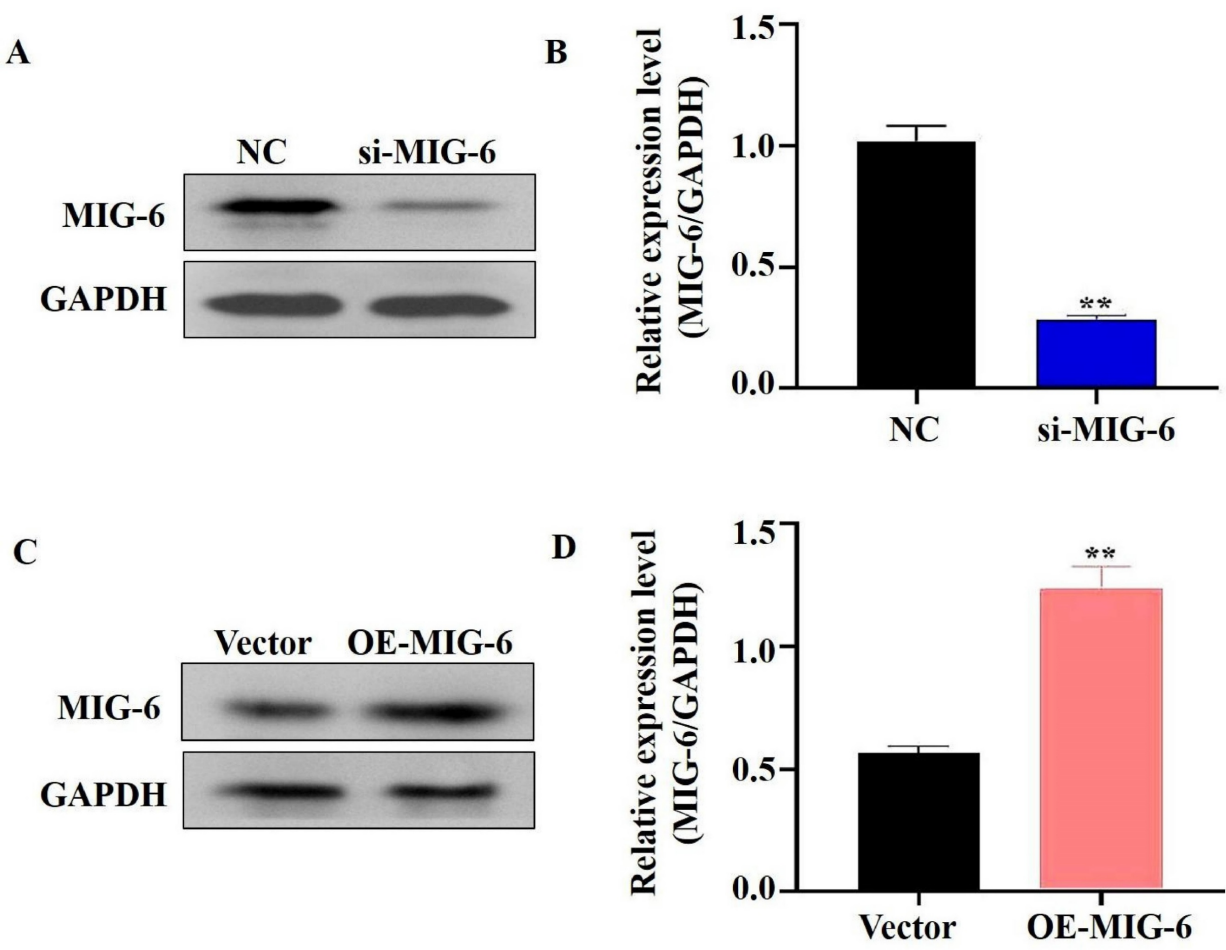
The expression of MIG-6 was enhanced in BGC-823 cells and knocked down in SGC-7901 cells. **(A)** and **(B)** The expression of MIG-6 in SGC-7901 cells transfected with si-MIG-6 decreased significantly. **P < 0.01 versus SGC-7901 cells transfected with NC SGC-7901 cells. **(C)** and **(D)** pcDNA3.0-MIG-6 could efficiently increase the expression of MIG-6 in BGC-823 cells. **P < 0.01 versus blank BGC-823 cells.

**Figure 3:**
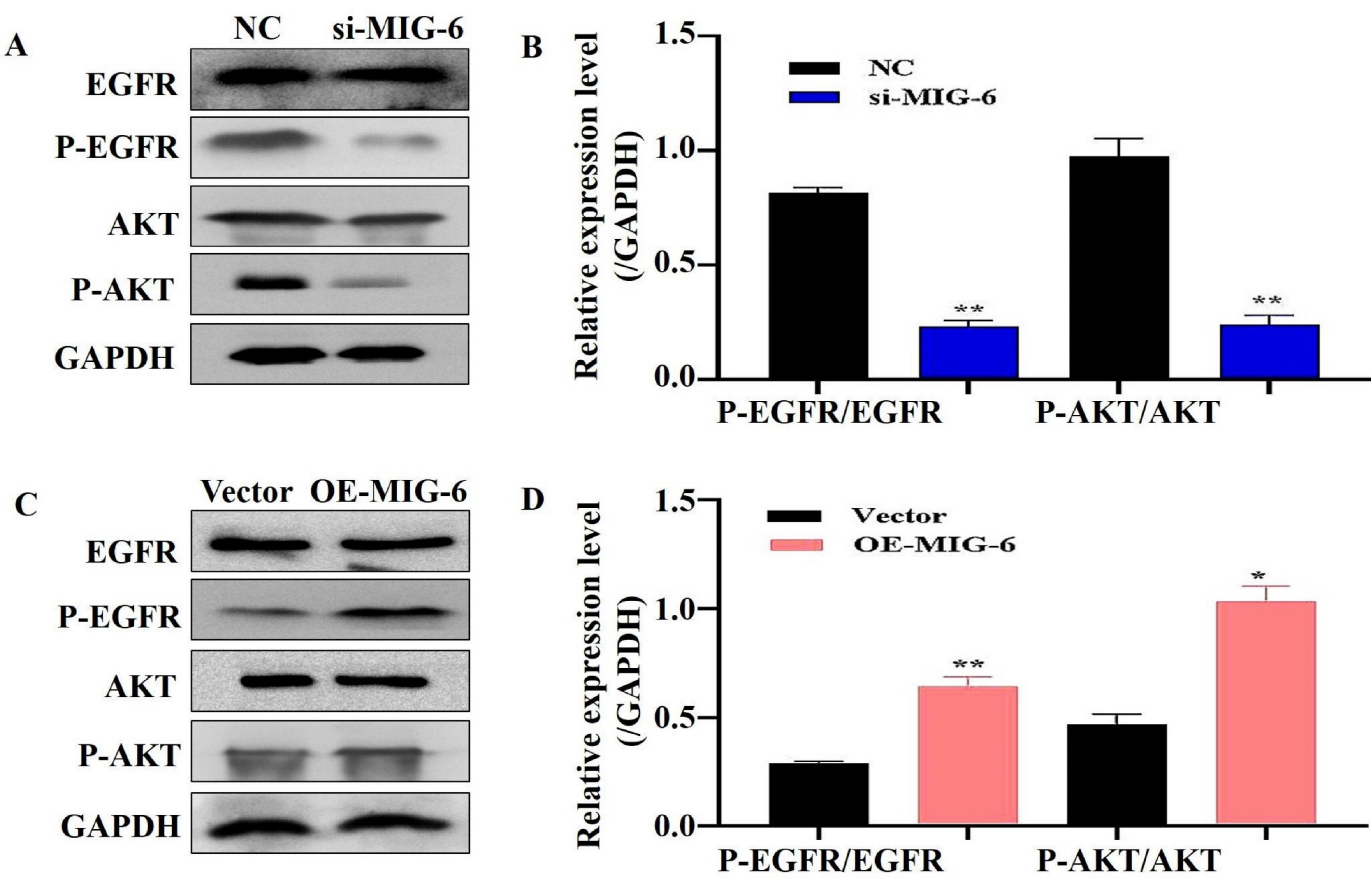
The regulation of MIG-6 in the EGFR/AKT pathway. **(A)** and **(B)** The knockdown of MIG-6 inhibits EGFR and AKT phosphorylation expression in SGC-7901 cells. **(C)** and **(D)** The overexpression of MIG-6 promotes EGFR and AKT phosphorylation expression in BGC-823 cells, *P < 0.05, **P < 0.01.

EGFR plays a critical role in cancer inhibition and growth 13. *MIG-6* suppresses tumor growth by blocking EGFR and its downstream signaling pathways 14. The findings indicated that *MIG-6* expression was knocked down in SGC-7901 cells (Figure 2A and B) and that the expressions of P-EGFR and P-AKT were decreased in SGC-7901 cells (Figure 3A and B). *MIG-6* was subsequently overexpressed in BGC-823 cells (Figure 2C and D). Furthermore, *MIG-6* overexpression induced the expressions of P-EGFR and P-AKT in BGC-823 cells (Figure 3C and D). These data demonstrate that *MIG-6* regulates EGFR activation and signaling in GC cells.

### 3.4 Aberrant MIG-6 expression influences the proliferation of GC cells

**Figure 4:**
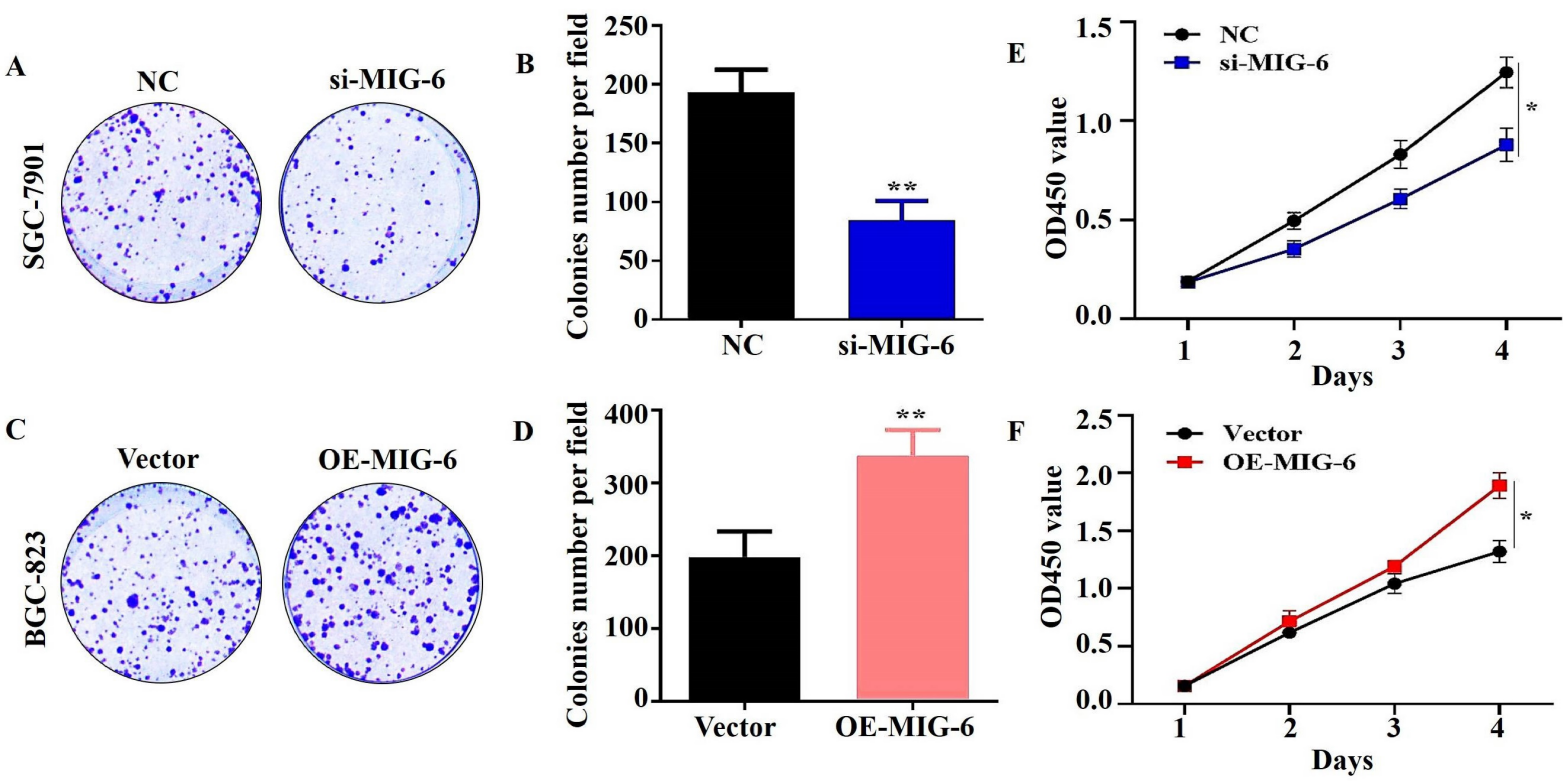
The knockdown of MIG-6 reduced the ability of proliferation of SGC-7901 cells **(A)** and **(B)** Colony formation assay was used to detect the ability of proliferation in SGC-7901 cells after MIG-6 knockdown. **(C)** and **(D)** Colony formation assay was performed to detect the ability of proliferation in BGC-823 cells with MIG-6 overexpression. **(E)** and **(F)** CCK-8 assay was performed in BGC-823 and SGC-7901 cells to examine the cell proliferation ability (Scale bar = 100 μm), *P < 0.05, **P < 0.01.

The results of the CCK-8 assay showed that the knockdown of *MIG-6* drastically reduced GC cell growth and colony formation (Figure 4A and E). Compared with the vector group, the proliferation of BGC-823 cells was enhanced in the OE-*MIG-6* group (Fig. 4F) and it formed larger and a greater number of clones (Figure 4C). Overall, these findings illustrate that *MIG-6* expression could promote GC cell proliferation *in vitro*.

### 3.5 MIG-6 overexpression induces EMT in GC cells

**Figure 5:**
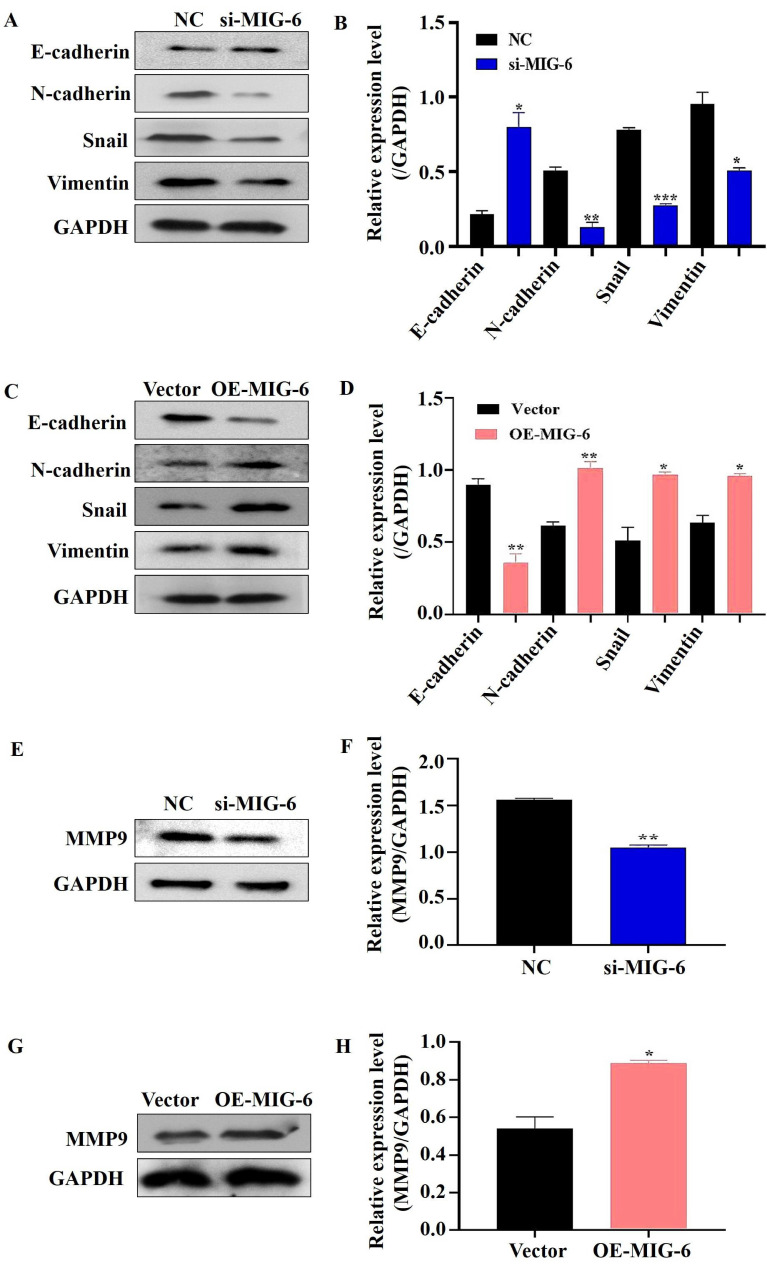
The expression of MIG-6 affected EMT-associated proteins in BGC-823 and SGC-7901 cells. **(A)** and **(B)** Western blotting revealed EMT phenotype with reduced Snail, Vimentin, and N-cadherin and boosted the E-cadherin expression in SGC-7901 cells transfected with si-MIG-6. **(C)** and **(D)** EMT phenotype displayed that high MIG-6 expression could induce Snail, Vimentin, and N-cadherin expression and reduce the E-cadherin expression. **(E)** and **(F)** The expression of MMP9 was decreased by western blotting in SGC-7901 cells transfected with si-MIG-6. **(G)** and **(H)** The expression of MMP9 was increased by western blotting in BGC-823 cells transfected with pcDNA3.0-MIG-6. *P < 0.05, **P < 0.01, ***P < 0.001.

A developmental and physical transformation known as EMT occurs before epithelial cell metastasis and invasion 15. The phenotype, shape, and polarity of epithelial cells change, and stromal cell features are simultaneously acquired 16. *MIG-6* downregulation decreased the expressions of N-cadherin, snail, and vimentin in SGC-7901 cells, but the epithelial marker E-cadherin increased drastically (Figure 5A and B). The overexpression of *MIG-6*, on the contrary, led to a modest decrease in E-cadherin and an increase in N-cadherin, snail, and vimentin expressions in BGC-823 cells (Figure 5C and D). Overall, these data suggest that aberrant *MIG-6* expression modulates EMT, which in turn modifies GC cell migratory capacity *in vitro*.

### 3.6 Aberrant MIG-6 expression influences cell metastasis *in vitro*

**Figure 6:**
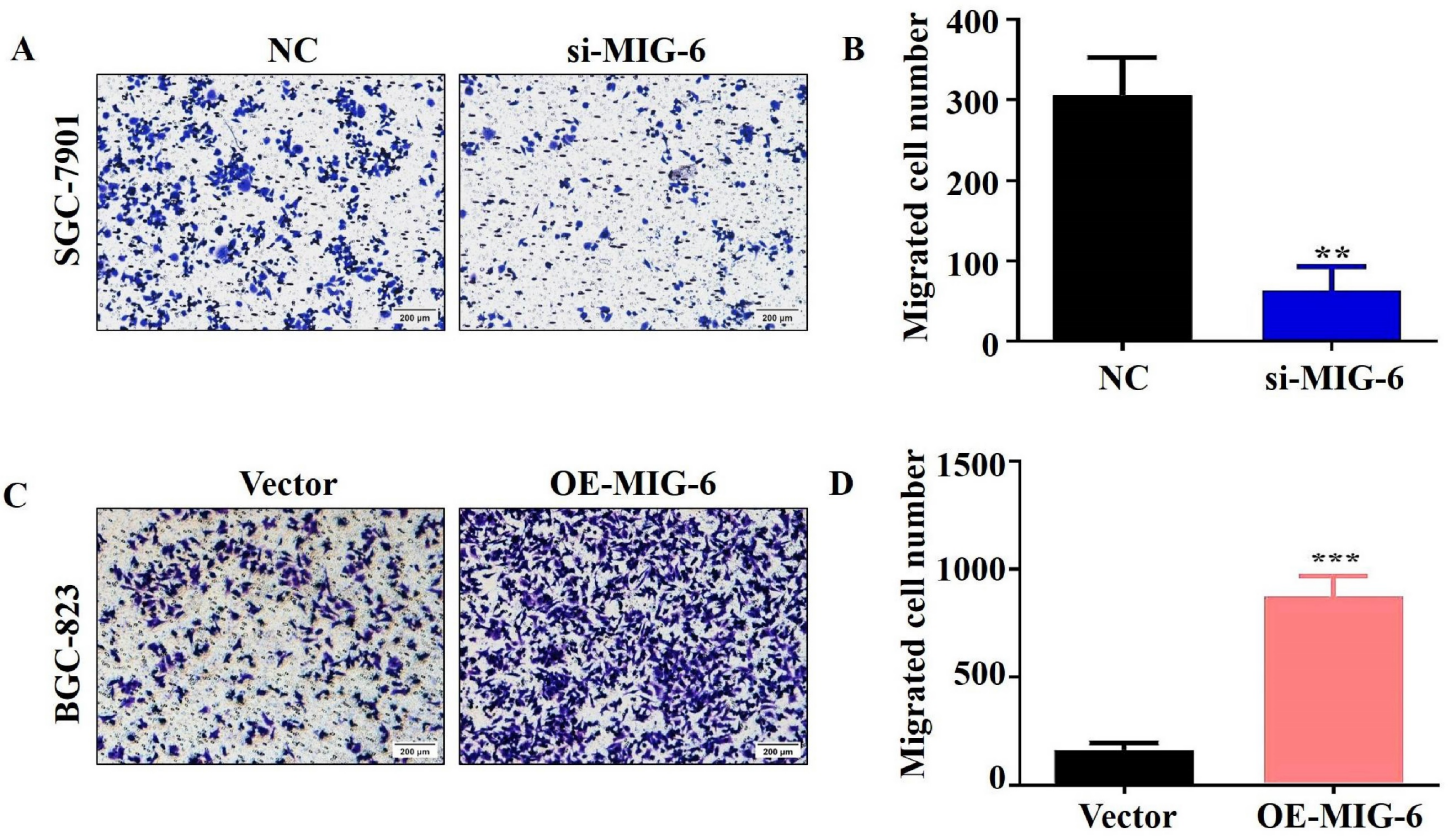
The migratory ability was evaluated by using transwell migration assay in BGC-823 and SGC-7901 cells. **(A)** and **(B)** The knockdown of MIG-6 reduced the migratory ability of SGC-7901 cells. **(C)** and **(D)** The overexpression of MIG-6 enhanced the migratory ability of BGC-823 cells. (200× magnification) **P < 0.01.

The transwell migration experiment showed that compared with the NC group, the migration of the SGC-7901 cells was dramatically decreased in the si-*MIG-6* group (Figure 6A and B). *MIG-6* overexpression greatly increased the number of migratory BGC-823 cells (Figure 6C and D). Owing to their proclivity to degrade the extracellular matrix, matrix metalloproteinases (MMPs) are assumed to play an important role in tumor invasion and metastasis in several cancers 17. Therefore, the expression of the migratory marker MMP9 was identified using western blotting. MMP9 expression was significantly reduced in SGC-7901 cells transfected with si-*MIG-6* (Figure 5E and F). MMP9 was significantly higher in *MIG-6*-overexpressing cells than in vector groups (Figure 5G and H). Collectively, our findings suggest that *MIG-6* regulates GC cell motility *in vitro*.

## 4. Discussion

*MIG-6* is a multiadapter protein known for its interaction with and negative regulation of EGFR. *MIG-6*-deficient mice exhibit hyperactivation of EGFR signaling and skin hyperplasia, and are highly prone to tumor formation in skin, lung, and other tissues. Chang et al. 18 compared multiple erlotinib-resistant cancer cell lines with their paired erlotinib-sensitive lines and showed that *MIG-6* expression is significantly increased in erlotinib-resistant cell lines. The observed *MIG-6* overexpression in erlotinib-resistant cells was linked to a decrease in EGFR activity and activation of AKT. These findings imply that *MIG-6* plays contradictory roles in different tumor environments. *MIG-6* acts as a tumor suppressor and is genetically altered or transcriptionally inhibited in lung cancer and glioblastoma. In these instances, constitutively active forms of EGFR promote tumor growth 9,10. Nonetheless, *MIG-6* deletions are rare in GC; hence, more investigations into *MIG-6* are warranted. This study observed that the expression of *MIG-6* was significantly increased in GC tissues and cells compared with normal controls. In addition, histological studies revealed that *MIG-6* expression in GC tissues contributes to disease progression and poor prognosis, uncovering a previously under-recognized tumor-promoting role of MIG-6 in cancer.

The expression of *MIG-6* has been demonstrated to be suppressed in nonsmall-cell lung cancer tissues and liver cancer, leading to increased EGFR/AKT signaling and enhanced cell proliferation and metastasis 8,19. However, this study found that *MIG-6* upregulation in GC tissue and cells could regulate the EGFR/AKT pathway and promote proliferation and metastasis, thereby resulting in unanticipated protumor outcomes. *MIG-6* interacts with EGFR via an ErbB-binding region in its C terminus. Biochemical studies on *MIG-6* uncovered that a 77 amino acid-region (336-412) is responsible for EGFR inhibition 20. Functional investigation of this area revealed the presence of an X-ray crystal structure (*MIG-6* residues 336-364, known as segment 1) that facilitates EGFR binding and partially suppresses EGFR by preventing the formation of asymmetric dimers. However, for EGFR suppression to be entirely effective *in vitro* and cells, 50 additional residues (residues 365-412, known as segment 2) must be present 7, 21. The inhibitory effect of *MIG-6* is controlled by EGFR-mediated Y394 phosphorylation, which is preceded by Src-mediated Y395 22. A study has reported that *MIG-6* is an activity-based EGFR inhibitor and that its selectivity for active EGFR is determined by EGFR-mediated *MIG-6* phosphorylation at Tyr394 22. Hence, we hypothesized that Y394 phosphorylation of *MIG-6* was defective in GC cells, which affected the capacity of *MIG-6* to decrease EGFR. However, additional studies are required to test this notion.

The EMT is a biological process in which cells lose their epithelial properties and instead acquire mesenchymal characteristics. Tumor initiation, malignant growth, tumor stemness, tumor cell motility, blood intravasation, metastasis, and treatment resistance have been documented to be related to EMT 15, 23. The activity of adherens junctions is dramatically altered during EMT, which could be mainly attributed to the replacement of E-cadherin by N-cadherin, a process known as “cadherin switching” 24. According to Chen et al. 25, vimentin expression is directly connected to poor clinical outcomes in breast cancer. Our study found that E-cadherin expression was reduced in BGC-823 cells overexpressing *MIG-6* although MMP9, N-cadherin, snail, and vimentin expressions were elevated. When siRNA was applied to SGC-7901 cells, E-cadherin increased but MMP9, N-cadherin, snail, and vimentin decreased. This result suggests that the expression of *MIG-6* may induce EMT and facilitate the development of GC and its metastasis. Nevertheless, the underlying mechanisms are yet to be elucidated. Further studies could focus on the precise mechanism by which *MIG-6* governs GC cell metastasis and invasion during tumor growth, particularly *in vivo*.

In conclusion, this study identified that enhanced *MIG-6* expression drives GC cell metastasis, proliferation, and EMT and contributes to disease progression in terms of tumor clinical stage and differentiation. The Kaplan-Meier plotter demonstrated that patients with GC exhibiting high *MIG-6* expression had a significantly shorter survival. Furthermore, our findings indicated that *MIG-6* may regulate the EGFR/AKT pathway and promote tumor growth and metastasis. In addition, our data showed that reducing *MIG-6* inhibits tumor development in GC, thus revealing a hitherto unknown prosurvival role of *MIG-6* in GC. These findings are encouraging as they suggest that *MIG-6* has the potential to be used as a diagnostic and prognostic biomarker in GC.

## Figures and Tables

**Table 1:** The correlation of MIG-6 expression with the clinical features of gastric cancer (GC)

Characteristics	n	High MIG-6 (n = 34)	Low MIG-6 (n = 19)	P-value
Gender				NS
Male	43	28	15	
Female	10	6	4	
Age (years)				0.20
≥60	38	22	16	
<60	15	12	3	
Tumor differentiation				**0.02**
Poor or moderate	41	30	11	
Well	12	4	8	
Primary tumor size (cm)				0.13
≥5	24	18	6	
<5	29	16	13	
Clinical stage				**0.02**
I or II	30	15	15	
III or IV	23	19	4	
Invasion depth				0.37
T1-2	13	7	6	
T3-4	40	27	13	
Lymph node metastasis (N factor)				0.40
Absent (N0)	21	15	6	
Present (N1-N3)	32	19	13	

P < 0.05 are indicated in bold

## References

[1] Sung H, Ferlay J, Siegel RL, Laversanne M, Soerjomataram I, Jemal A (2021). Global Cancer Statistics 2020: GLOBOCAN Estimates of Incidence and Mortality Worldwide for 36 Cancers in 185 Countries. CA Cancer J Clin.

[2] Smyth EC, Nilsson M, Grabsch HI, van Grieken NC, Lordick F (2020). Gastric cancer. Lancet.

[3] Lordick F, Carneiro F, Cascinu S, Fleitas T, Haustermans K, Piessen G (2022). Gastric cancer: ESMO Clinical Practice Guideline for diagnosis, treatment and follow-up. Ann Oncol.

[4] Wang Q, Chen C, Ding Q, Zhao Y, Wang Z, Chen J (2020). METTL3-mediated m(6)A modification of HDGF mRNA promotes gastric cancer progression and has prognostic significance. Gut.

[5] Kim TH, Lee DK, Cho SN, Orvis GD, Behringer RR, Lydon JP (2013). Critical tumor suppressor function mediated by epithelial Mig-6 in endometrial cancer. Cancer Res.

[6] Frosi Y, Anastasi S, Ballarò C, Varsano G, Castellani L, Maspero E (2010). A two-tiered mechanism of EGFR inhibition by RALT/MIG-6 via kinase suppression and receptor degradation. J Cell Biol.

[7] Zhang X, Pickin KA, Bose R, Jura N, Cole PA, Kuriyan J (2007). Inhibition of the EGF receptor by binding of MIG-6 to an activating kinase domain interface. Nature.

[8] Reschke M, Ferby I, Stepniak E, Seitzer N, Horst D, Wagner EF (2010). Mitogen-inducible gene-6 is a negative regulator of epidermal growth factor receptor signaling in hepatocytes and human hepatocellular carcinoma. Hepatology.

[9] Maity TK, Venugopalan A, Linnoila I, Cultraro CM, Giannakou A, Nemati R (2015). Loss of MIG-6 Accelerates Initiation and Progression of Mutant Epidermal Growth Factor Receptor-Driven Lung Adenocarcinoma. Cancer Discov.

[10] Ying H, Zheng H, Scott K, Wiedemeyer R, Yan H, Lim C (2010). Mig-6 controls EGFR trafficking and suppresses gliomagenesis. Proc Natl Acad Sci U S A.

[11] He J, Li CF, Lee HJ, Shin DH, Chern YJ, Pereira De Carvalho B (2021). MIG-6 is essential for promoting glucose metabolic reprogramming and tumor growth in triple-negative breast cancer. EMBO Rep.

[12] Kang DH, Jung SS, Yeo MK, Lee DH, Yoo G, Cho SY (2020). Suppression of Mig-6 overcomes the acquired EGFR-TKI resistance of lung adenocarcinoma. BMC Cancer.

[13] Harrison PT, Vyse S, Huang PH (2020). Rare epidermal growth factor receptor (EGFR) mutations in non-small cell lung cancer. Semin Cancer Biol.

[14] Cheng WL, Feng PH, Lee KY, Chen KY, Sun WL, Van Hiep N (2021). The Role of EREG/EGFR Pathway in Tumor Progression. Int J Mol Sci.

[15] Voulgari A, Pintzas A (2009). Epithelial-mesenchymal transition in cancer metastasis: mechanisms, markers and strategies to overcome drug resistance in the clinic. Biochim Biophys Acta.

[16] Greenburg G, Hay ED (1982). Epithelia suspended in collagen gels can lose polarity and express characteristics of migrating mesenchymal cells. J Cell Biol.

[17] Yang S, Liu Y, Li MY, Ng CSH, Yang SL, Wang S (2017). FOXP3 promotes tumor growth and metastasis by activating Wnt/β-catenin signaling pathway and EMT in non-small cell lung cancer. Mol Cancer.

[18] Chang X, Izumchenko E, Solis LM, Kim MS, Chatterjee A, Ling S (2013). The relative expression of Mig-6 and EGFR is associated with resistance to EGFR kinase inhibitors. PloS one.

[19] Li Z, Dong Q Fau - Wang Y, Wang Y Fau - Qu L, Qu L Fau - Qiu X, Qiu X Fau - Wang E, Wang E (2012). Downregulation of Mig-6 in nonsmall-cell lung cancer is associated with EGFR signaling. Mol Carcinog.

[20] Wang Z, Raines LL, Hooy RM, Roberson H, Leahy DJ, Cole PA (2013). Tyrosine phosphorylation of mig6 reduces its inhibition of the epidermal growth factor receptor. ACS Chem Biol.

[21] Wang Z, Longo PA, Tarrant MK, Kim K, Head S, Leahy DJ (2011). Mechanistic insights into the activation of oncogenic forms of EGF receptor. Nat Struct Mol Biol.

[22] Park E, Kim N, Ficarro SB, Zhang Y, Lee BI, Cho A (2015). Structure and mechanism of activity-based inhibition of the EGF receptor by Mig6. Nat Struct Mol Biol.

[23] Pastushenko I, Blanpain C (2019). EMT Transition States during Tumor Progression and Metastasis. Trends Cell Biol.

[24] Cavallaro U, Schaffhauser B, Christofori G (2002). Cadherins and the tumour progression: is it all in a switch?. Cancer Lett.

[25] Chen Z, Fang Z, Ma J (2021). Regulatory mechanisms and clinical significance of vimentin in breast cancer. Biomed Pharmacother.

